# Effect of experimental laser imperfections on laser wakefield acceleration and betatron source

**DOI:** 10.1038/srep27846

**Published:** 2016-06-21

**Authors:** J. Ferri, X. Davoine, S. Fourmaux, J. C. Kieffer, S. Corde, K. Ta Phuoc, A. Lifschitz

**Affiliations:** 1 CEA, DAM, DIF, 91297 Arpajon, France; 2 Laboratoire d’Optique Appliquée, ENSTA, CNRS, Ecole Polytechnique, UMR 7639, 91761 Palaiseau, France; 3 INRS-EMT, Université du Québec, 1650 Lionel Boulet, Varennes J3X 1S2, Québec, Canada

## Abstract

Laser pulses in current ultra-short TW systems are far from being ideal Gaussian beams. The influence of the presence of non-Gaussian features of the laser pulse is investigated here from experiments and 3D Particle-in-Cell simulations. Both the experimental intensity distribution and wavefront are used as input in the simulations. It is shown that a quantitative agreement between experimental data and simulations requires to use realistic pulse features. Moreover, some trends found in the experiments, such as the growing of the X-ray signal with the plasma length, can only be retrieved in simulations with realistic pulses. The performances on the electron acceleration and the synchrotron X-ray emission are strongly degraded by these non-Gaussian features, even keeping constant the total laser energy. A drop on the X-ray photon number by one order of magnitude was found. This clearly put forward the limitation of using a Gaussian beam in the simulations.

Since the theoretical work of Tajima and Dawson in 1979^1^, laser wakefield acceleration (LWFA) has been established as an efficient and compact technique for accelerating particles. In 2004 the first multi-MeV quasi-monoenergetic electron bunches^2^,^3^,^4^ were obtained. Nowadays, the use of the bubble regime^5^ allows to routinely generate multi-GeV electrons^6^,^7^,^8^,^9^. Besides, the reliability of the experimental techniques has improved, leading to a better shot-to-shot reproducibility for the beam charge and energy^10^,^11^,^12^.

The improvement in the understanding and control of the electron acceleration was accompanied with the development of associated radiation sources. In particular the electrons wiggle during the acceleration process due to the radial electric fields in the wakefield. This transverse motion of the electrons, called betatron motion, generates a synchrotron-type radiation, often referred as betatron emission. This source, characterized by a small source size and a broadband spectrum in the keV to tens of keV range^13^, has been used to realize high-resolution phase contrast imaging^14^,^15^, and seems to be a promising source for different domains, such as biology, medical imaging, etc.^16^.

However, further improvement of the electron and X-ray beam properties and shot-to-shot stability is still mandatory for most of the societal applications of this technology. One of the key parameters affecting these features is the quality of the laser spot. In a very recent study, a laser halo was shown to strongly degrade the electron parameters^17^. In addition with the transverse intensity distribution, distortion of the wavefront in the focal plane, such as comatic aberrations, can influence the beam emittance and the X-ray emission^18^,^19^. Besides, the laser spot in the focal plane does not contain all necessary information as the focusing geometry can lead to laser filamentation and poor performances^20^,^21^. In some recent experiments performed with a high repetition-low energy laser system, the wavefront was optimized with respect to the electron beam quality instead of the focal spot quality^22^. It was found that the optimal electron quality did not correspond to the optimal focal spot, suggesting that the pulse properties out of the focal plane are an important ingredient of the accelerator.

Most theoretical and numerical studies on laser-plasma electron acceleration deal with ideal Gaussian or Laguerre-Gaussian transverse intensity profiles^23^. In some works, more realistic pulse distributions are used, which fit experimentally measured laser spots^24^. In the present work we perform simulations using not only the experimental focal spot but also the experimental wavefront. A good qualitative and quantitative agreement between the experimental data and the simulations was only achieved when both features were taken into account. We show that in that case the performances of the accelerator are drastically degraded in comparison to the ideal Gaussian case. The energy of the X-ray source can be reduced by one order of magnitude. These improved simulations allow us also to enlighten the origin of some trends found in the experiment.

The structure of the paper is as follows. We first start by describing the experimental and numerical settings and by discussing the inhomogeneities of the experimental laser pulse. Then we compare the simulation results on the laser propagation and focusing as well as the electron injection and acceleration processes with Gaussian pulses or with realistic pulses. Finally we highlight the influence of the laser spot features on the X-ray emission.

## Results

### Numerical modeling and phase reconstruction

In an experiment performed on the 800 nm Ti:Sapphire laser system at the Advanced Laser Light Source (ALLS) facility at INRS-EMT, a 30 fs FWHM and 2.5 J linearly polarized laser is focused on a 18 *μ*m FWHM focal spot at the entrance of an helium gas nozzle. Different nozzle lengths are used, ranging from 3 mm to 7 mm, but the electron density is kept close to *n*_*e*_ = 6 × 10^18^ cm^−3^. For each length, the focal plane position is changed to maximize the X-ray signal. To determine the maximum electron energy and the electron charge, an electron spectrometer with a low energy cut-off at 140 MeV is used. Some details on the gas profiles for each nozzle as well as the main electron beam and X-ray properties are summed up in Table 1 (see Methods). The experimental results are averaged over 10 shots due to shot-to-shot variation of the electron and X-ray spectra. Note that all the experimental parameters are measured simultaneously for each laser shot. Experimental results exhibit an increase of the number of emitted photons with the nozzle length, even if this one is much longer than the theoretical depletion length *L*_*d*_ ~ 2.6 mm and dephasing length *L*_*p*_ ~ 1.3 mm.

To understand this counter-intuitive result, we have performed 3D PIC simulations with the code CALDER^25^ (see Methods). The laser beam is always described by a 30 fs FWHM pulse with a linear polarization. However, three different options are chosen to simulate its transverse profile: (1) a gaussian laser beam is used with a focal spot *w*_0_ = 18 *μ*m leading to a normalized potential vector *a*_0_ = 2.32 (matching the experimental value of 1.35 J contained in the useful focal spot). (2) The transverse profile measured experimentally in the focal plane is used and we assume the phase to be null in this plane. (3) The transverse profile measured experimentally in the focal plane is used and the reconstructed experimental phase is also used. In the following, these three cases are referred as the cases with (1) a gaussian beam, (2) the experimental spot only and (3) the experimental spot and phase. These three laser beams contains the same energy. The transverse profiles being different, the laser intensity peak can also be different in these three cases. So unlike in the previous work in which the laser peak intensity and focal spot size were modified when high order Laguerre-Gaussian profiles were used^23^, in our case the main pulse parameters (energy, duration and waist) are kept close to each other. Only the complex laser imperfections can explain the differences in the electron and X-ray beam results that will be presented in this paper.

**Figure 1:**
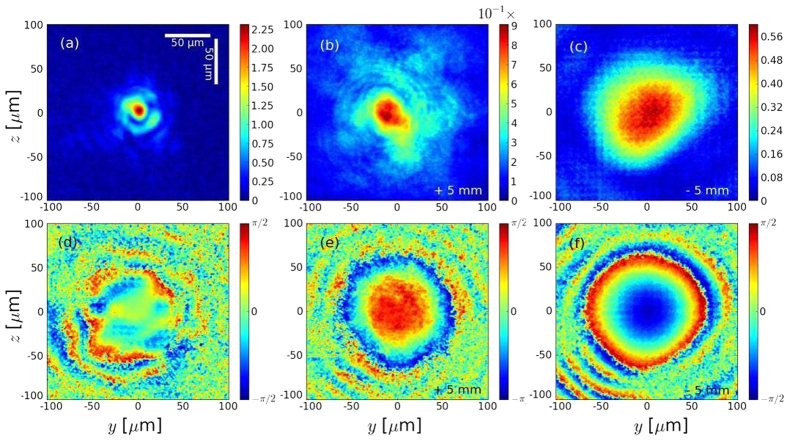
(**a**,**b**) are the laser spots (normalized potential vector) measured experimentally at the focal plane and 5 mm after respectively. (**d**,**e**) are the phases reconstructed with the GSA corresponding to (**a**,**b**) respectively. Figure (c) (resp. (**f** )) shows *a*_0_ for the focal spot (resp. the phase) of the laser 5 mm before the focal plane and calculated with the GSA.

The reconstruction of the phase is performed using the Gerchberg-Saxton algorithm^26^ (GSA), which can calculate the phase and the spot shape of the laser at any point from at least two images of the laser spot in different planes close enough to the focal plane. This algorithm has already been used to reconstruct the phase in LWFA with mJ laser^27^. In our case, the validity of the algorithm was checked on a gaussian beam with satisfactory results: if two intensity profiles in two different planes are given as an input, the algorithm can reconstruct the intensity and phase profiles in any plane with a good agreement with the theoretical gaussian beam profiles. Experimentally, the laser spot was measured at two positions on the same laser shot: in the focal plane and 5 mm after the focal plane. In Fig. 1a,b we show *a*_0_ for these two laser spots. The GSA was used with these two measured spots as an input and, as an example, we show the reconstructed phase at and 5 mm after the focal plane (Fig. 1d,e), as well as the reconstructed phase and spot 5 mm before the focal plane (Fig. 1c,f).

**Figure 2:**
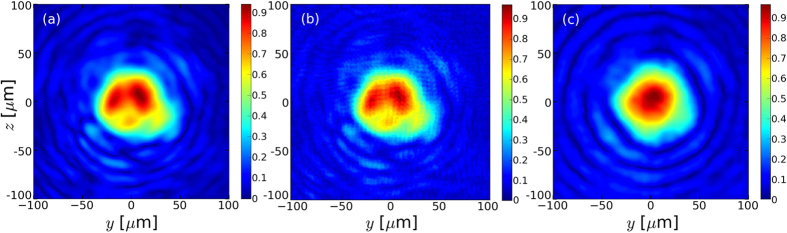
Laser spots (normalized potential vector) after a propagation in vacuum on 2*Z*_*R*_ (1.8 mm) from the focal plane calculated by (**a**) Particle-in-Cell using the reconstructed wavefront, (**b**) Gerchberg-Saxton and (**c**) Particle-in-Cell using a flat wavefront.

To further test the algorithm, the propagation in vacuum of the laser with the experimental spot and phase was simulated with CALDER, and the results were compared with the GSA output. The simulation starts with the laser in the focal plane and results are compared after 2 Rayleigh length (*Z*_*R*_ = 0.92 mm) of propagation. A very good agreement is found (Fig. 2a,b). For comparison, assuming a null phase at the focal plane leads to a different result after 2*Z*_*R*_ (Fig. 2c). As can be seen comparing 2a,c, the use of a realistic wavefront instead of a flat one results in a more complex intensity pattern out of the focal plane. Then, the use of the experimental spot and a flat wavefront or of a Gaussian pulse tends to smooth the laser intensity profile in a significant fraction of the interaction region. As we will see, this smoothing has important consequences over the laser propagation and the acceleration processes.

### Laser focusing and electron acceleration

To reproduce the experiment, we run simulations with the parameters given in Table 1. The plasma consists in a plateau whose length varies between 3.1 mm and 7.5 mm, with linear gradients at the entrance and the exit. The position of the focal plane compared with the beginning of the density plateau is also indicated in Table 1. In the experiments, the focal plane position is determined after scanning along the laser propagation axis in order to get the maximal X-ray signal. The X-ray output was optimized when the focal plane was displaced further in the plasma for the longer nozzles. In order to understand this, we first investigate the focusing effect for the different laser profiles.

**Figure 3:**
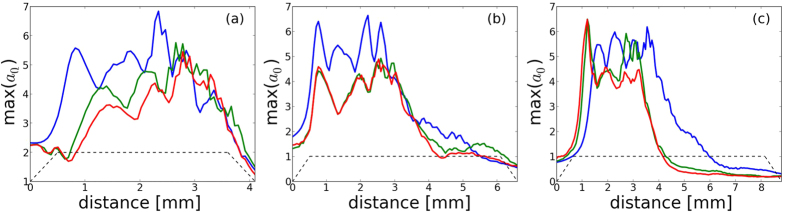
Evolution of the normalized peak of the potential vector for the 3 mm (**a**), 5 mm (**b**) and 7 mm nozzle (**c**). In blue, with a gaussian laser; in green with the experimental spot only; in red with the experimental spot and phase. The dashed black lines correspond to the plasma profiles.

The evolution of the maximum of the vector potential *a*_0_ is shown in Fig. 3 for the 3 mm, 5 mm and 7 mm nozzles. In all cases, the maximal intensity first increases when the laser self-focuses at the entrance of the plasma. Then it modulates during about 3 mm, corresponding to successive self-focusing and defocusing periods of the laser during its propagation. At the end, the maximal value of *a*_0_ decreases which indicates that the laser energy is depleted and the laser is no longer self-focused.

**Figure 4:**
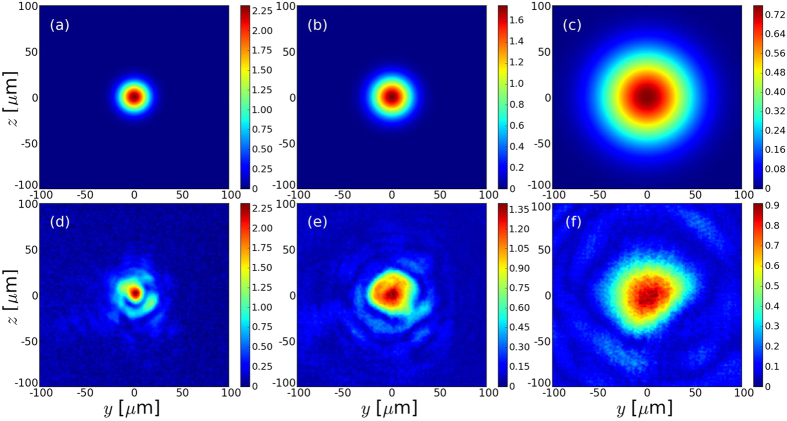
Laser spots (normalized potential vector) at the beginning of the simulation with the gaussian laser for the 3 mm (**a**), 5 mm (**b**) and 7 mm (**c**) nozzles. (**d**–**f** ) are the spots at the beginning of the simulation with the experimental spot and phase for the same nozzles (resp. 3 mm, 5 mm and 7 mm).

For the 3 mm nozzle, we can see (Fig. 3a) that the maximal intensity reached by the laser is lower when the laser is not gaussian. The energy in the ring, out of the center spot, is actually not efficiently self-focused, resulting in a self-guided pulse which actually contains a lower energy and reaches a lower intensity. Because of the lower intensity, the conversion of the laser energy into the wakefield is less efficient with a more realistic laser^28^. The other remarkable fact is that it takes a longer time for the laser to self-focus when it is not ideal. Due to the inhomogeneities of the incoming laser and the lower effective energy (Fig. 4d, to be compared with Fig. 4a), the laser with the experimental spot and phase will be fully self-focused 1 mm after the gaussian beam. The realistic laser first homogenizes during 1 mm in the plasma due to laser-plasma interaction before it can properly self-focus. As a consequence, the physical processes involved (such as bubble formation and electron acceleration) will be delayed by this length. Note that the length required to self-focus is not negligible for a 3 mm gas jet, therefore electron acceleration is likely to be significantly shortened. On the contrary with the 5 mm nozzle (Fig. 3b), the self-focusing process begin approximately at the same time but the maximal intensity is lower when the experimental profile is used. The similar timing for the self-focusing is due to a much more homogeneous laser spot before focus (Fig. 4e, to be compared with Fig. 4b), but the spot inhomogeneities still lead to a lower maximal intensity because of the fact that a fraction of the laser energy is actually not self-focused. This is even more striking for the 7 mm nozzle, where the maximal *a*_0_ reached is about the same for all lasers. In this last case, we can expect the gaussian beam to be a good enough approximation of the experimental beam.

**Figure 5:**
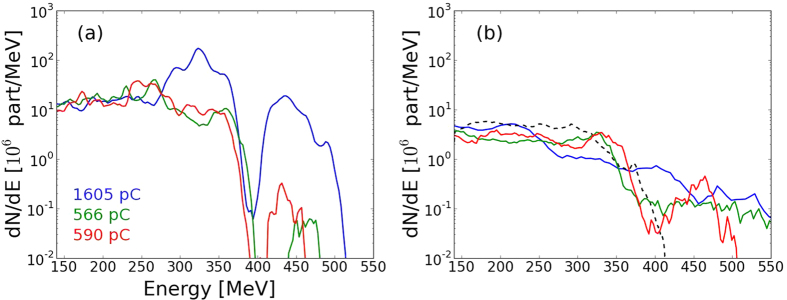
(**a**) (resp. (**b**)) Electron distribution for the 5 mm nozzle after 3.5 mm (resp. 6.6 mm) of propagation. In blue, with a gaussian laser, in green with the experimental spot only and in red with the experimental spot and phase. In dashed black line, the experimental electron energy spectrum (averaged over ten shots).

The different focusing behavior has an influence on the electron acceleration. We focus here on the results with the 5 mm nozzle. The electron charge after 3.5 mm of propagation is shown in Fig. 5a. The total energy in the accelerated electron bunch is the highest at this position for the three laser beam cases. After this point, there is no more energy transfer from the laser to the electron bunch, and the electron bunch itself will start losing energy by creating its own wakefield. For the gaussian laser, the charge over 140 MeV is 1600 pC at 3.5 mm, with a high energy electron bunch close to 450 MeV. This charge is about three times higher than with lasers including experimental features (~550–600 pC). Besides, for these more realistic lasers, the high energy electron bunch at 450 MeV almost entirely vanishes and the quasi-monoenergetic structure of the acceleration disappears. Adding the imperfections of the experimental laser leads to a less efficient self-focusing and to the generation of a wakefield with a lower amplitude. As a consequence the energy transfer from the laser to the accelerated electrons reduces, even if the energy contained in the laser spot is the same. However after the laser depletion, most of the electron charge and energy is lost in the remaining part of the plasma due to the creation of the wakefield driven by the electron beam, so the difference in the electron distribution at the exit of the plasma is not much significant (Fig. 5b). For the 5 mm nozzle the final charges are 185 pC for the gaussian beam and 160 pC if the experimental spot and phase are used. Both cases are close to the 230 pC observed in the experiment at the exit of the target.

Physically, the 7 mm case is quite similar to the 5 mm one; it involves electron acceleration and then charge and energy loss after the laser depletion. However for the 3 mm case, the length of the plasma plateau is close to the theoretical laser depletion length. The acceleration of the electrons is stopped before the full laser depletion, and the final charge is much reduced when the experimental spot and phase are used: 475 pC instead of 1357 pC with a gaussian laser and 645 pC with the experimental spot only. This is much closer to the observed average experimental charge (202 pC).

### Laser inhomogeneities effects on the X-ray emission

**Figure 6:**
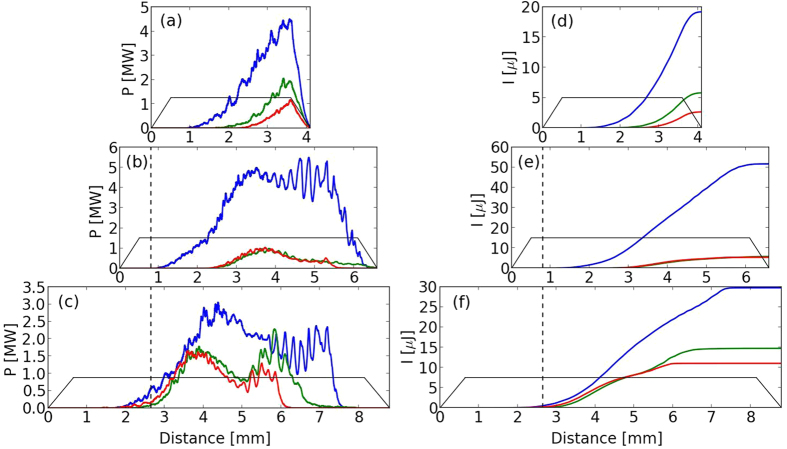
(**a**–**c**) Power *P*_*ray*_ (eq. (1)) radiated during the propagation for the 3 mm, 5 mm and 7 mm nozzles respectively. In blue, with a gaussian laser, in green with the experimental spot only and in red with the experimental spot and phase. (**d**–**f** ) The corresponding radiated energy *I*_*ray*_ (eq. (2)). The solid black lines correspond to the plasma profile and the dotted black lines mark the position of the focal plane.

The use of the real pulse features will also affect the synchrotron emission. Figure 6a–c shows the evolution of the radiated power *P*_*ray*_ for all nozzles depending on the laser beam used, where
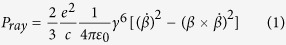
with *e* the electron charge, *c* the speed of light, *ε*_0_ the vacuum permittivity, *γ* the Lorentz factor of the electron, *β* and 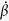 respectively the speed and acceleration of the electron normalized to *c*.

For the 5 and 7 mm nozzles, oscillations of *P*_*ray*_ are observed in the last few millimeters. They are triggered by the coherent transverse oscillations of the electron beam that occur due to carrier-enveloppe phase (CEP) effects^29^ which induce a transverse wiggling of the wakefield when the laser depletes. Then the acceleration regime slowly changes from a LWFA regime to a beam driven wakefield regime^30^. For the longest nozzles, once the laser is depleted the creation of the wakefield is ensured by the accelerated electron beam, and part of its tail is accelerated by the created fields. For the 7 mm nozzle, the X-ray energy emitted is still consequent once the beam driven wakefield phase has started.

For each nozzle, we can see that the radiated power strongly drops when the experimental laser spot is used and diminishes further when the phase information is added. In the case of the 3 and 5 mm nozzles, the total energy radiated during the acceleration (*I*_*ray*_) is about 1 order of magnitude lower (Fig. 6d,e), with *I*_*ray*_ given by:
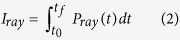


This big reduction for the 3 and 5 mm nozzles is linked to the less efficient and delayed self-focusing that occurs with the realistic laser in comparison with the gaussian beam (Fig. 3): the injection starts later and less charge is accelerated to high energy (Fig. 5a). As a result, the radiation emission is delayed and its power is strongly reduced (Fig. 6a,b). Figure 6a,b also explain the higher final energy radiated with the 5 mm nozzle than with the 3 mm nozzle: the emission is still important after 3.5 mm due to the transition to a beam driven wakefield regime. In the 7 mm nozzle case (Fig. 6c,f), the more similar self-focusing scenarios with the realistic laser or with a gaussian beam lead to closer results: the radiated power rises at the same time, its amplitude difference is lower and the final radiated energy is much closer (factor ~2 instead of 1 order of magnitude). The results with the realistic laser being much closer to the optimal (i.e. gaussian beam) case, the total radiated energy is therefore higher with this nozzle than with the 5 mm nozzle. This is not the case for the gaussian beam: the radiated power reached in the LWFA regime is smaller for the 7 mm nozzle (~3 MW) than for the 3 and 5 mm nozzles (~5 MW), which leads to a lower energy radiated with the 7 mm nozzle than with the 5 mm nozzle. This can be explained by the fact that the optimal focal plane position is close to the plasma entrance with the gaussian beam: an early focusing do not achieve a better homogeneity of the laser spot, while part of the energy is lost during the focusing in the plasma and is not used for particle acceleration.

From *I*_*ray*_, we have calculated the energy conversion efficiency from the laser to the accelerated electrons above 140 MeV *η*_*l*→*e*_ and from the electrons to the x-rays beam *η*_*e*→*x*_. For the 5 mm nozzle, we obtain *η*_*l*→*e*_ = 0.13 and *η*_*e*→*x*_ = 3 × 10^−4^ for the gaussian laser, while with the experimental spot and phase, the efficiencies are *η*_*l*→*e*_ = 0.045 and *η*_*e*→*x*_ = 9 × 10^−5^. Both values are lower for the realistic laser, as first less electrons are accelerated and secondly they get lower average energies and the energy scattered in the radiation scales as *γ*^2^. As a consequence, the total conversion efficiency *η*_*l*→*x*_ is one order lower with the realistic laser (*η*_*l*→*x*_ = 4.0 × 10^−6^ for *η*_*l*→*x*_ = 3.8 × 10^−5^ with the gaussian laser).

**Figure 7:**
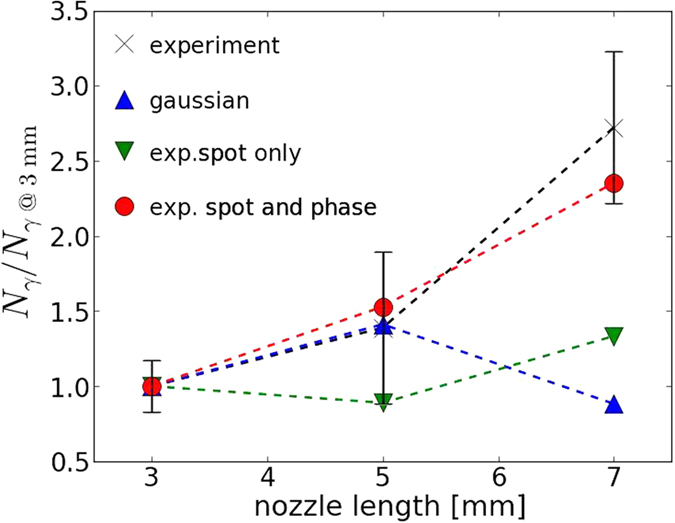
Number of photons as a function of the nozzle length. The black crosses are the experimental results and the blue triangles (resp. green triangles and red circles) the numerical results with the gaussian profile (resp. experimental spot and experimental spot and phase). These values are normalized to the number of photons obtained with the 3 mm nozzle with the same laser. Horizontal black lines represents the standard deviation for the experimental measures.

The simulated number of emitted photons *N*_*γ*_ emitted in the interval 0–30 keV is summarized in Table 2 (see Methods for the calculation of *N*_*γ*_). In all cases, *N*_*γ*_ is reduced when a realistic laser beam is used (Table 2). For the 3 mm nozzle the gaussian laser overestimates the number of photons by a factor ~6 when compared with the simulation with the realistic laser and still overestimates it by a factor >2 when the information on the phase is not accounted for. This is not so important for the 7 mm nozzle where this factor is ~2 instead of 6. This is due to the fact that the laser starts to interact with the plasma while the intensity distribution is still somehow homogeneous. Only the simulations using a realistic laser can reproduce the experimental observations of an increase of the photon number with a longer nozzle (Fig. 7). This is a surprising result as *L*_*d*_ and *L*_*p*_ are shorter than the nozzle length. Two main reasons can be deduced from the simulations: (i) the transition of a LWFA to a beam driven regime leads to X-ray emission even after the depletion length, which explains the photon number increase from the 3 mm to the 5 mm nozzle, (ii) starting the laser-plasma interaction before reaching the focal plane leads to the focalisation and propagation of a more homogeneous and efficient laser beam. However, the focal plane position delays the bubble formation and electron acceleration and is beneficial only if the plasma is long enough (nozzle ≥5 mm in our case). This second observation explains well the radiation increase from the 5 mm to the 7 mm nozzle if a realistic laser profile is used, but can not be reproduced and understood if only gaussian beams are used, which can be a source of misinterpretation of the experimental result.

In terms of number of photons, simulations using the experimental spot and phase are much closer to the experimental results given in Table 2. But even with this realistic laser, the number of photons are overestimated by a factor 4–5 compared with the average results of the experiment. Experimentally, the shot to shot fluctuation is important and this overestimation is well reduced when compared with the highest value obtained in the experiment (factor ~2 for all the nozzles). In addition, not only the number of photons but their energies are also modified. A critical energy *E*_*c*_ for the X-ray spectra is determined by fitting the spectra with theoretical synchrotron spectra on the interval 10–30 keV^31^. The synchrotron spectrum is given by the formula 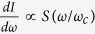 where 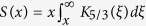, with *K*_5/3_ a modified Bessel function of the second kind. In the case of the 5 mm nozzle, the critical energy for the gaussian laser was calculated to be *E*_*c*_ = 18.1 keV whereas it is only 12.4 keV if the experimental spot and phase are used, much closer to the 12.3 keV experimental value. This reduction is due to a lower average energy obtained by the electrons during their acceleration if a realistic beam is used (the high energy bunch is nearly removed, cf Fig. 5a).

**Figure 8:**
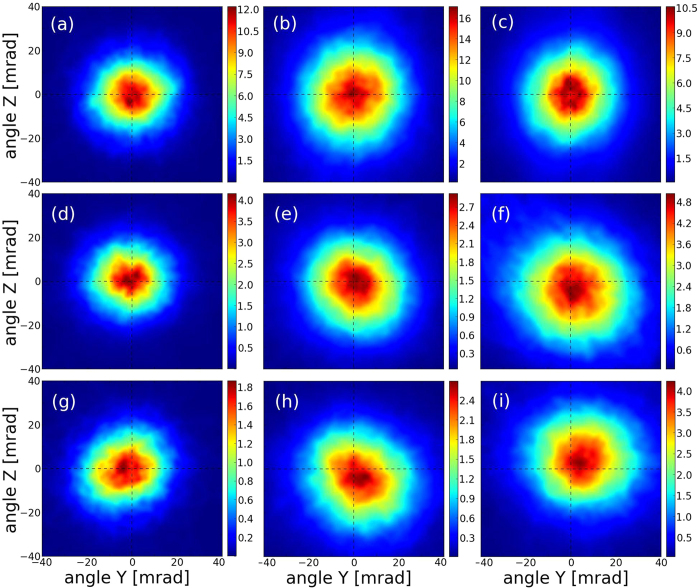
Intensity of the radiation per angle unit (mJ/mrad). (**a**–**c**) is the gaussian case, (**d**–**f** ) the case with the experimental spot only and (**g**–**i**) the case with the experimental spot and phase. (**a**,**d**,**g**) are results with the 3 mm nozzle, (**b**,**e**,**h**) results with the 5 mm nozzle and (**c**,**f**,**i**) results with the 7 mm one.

Finally the angle of emission is relatively stable with the different laser profiles (Fig. 8). The widening of the spots between the 3 mm nozzle and the 5 and 7 mm ones is explained by the wider motion acquired by the electrons due to the CEP effects and during the transition to a beam driven regime. However while the X-ray spot is centered on the incoming laser propagation axis if a gaussian laser is used, it tends to deviate from this axis with a more realistic laser profile, particularly when adding the information on the experimental phase. Deviation of the order of ~5–6 mrad are obtained. This pointing deviation is not linked to CEP effects because it does not particularly occur in the laser polarization plane: this behavior is clearly a consequence of the laser inhomogeneities that tends to deviate the laser from its initial axis of propagation once in the plasma. The direction of the deviation is quite stable along one simulation, but completely differs when the nozzle size is changed. So this kind of behavior is quite difficult to predict as for the same laser beam, simply modifying the position of the focal plane utterly changes the direction of the center of the X-ray emission. As the X-ray radiation represents a signature of the electron motion in the bubble^32^, we can deduce that laser inhomogeneities and in particular phase effects are susceptible to change the direction of emission of the electron beam.

## Discussion

In this paper we have shown the importance of taking into account the experimental imperfections of the laser in the simulations. Using a gaussian beam does not allow to fully reproduce the data such as the X-ray signal increase with the plasma length in the present case. In addition, using a gaussian beam can lead to misinterpretations: only the beam-driven wakefield regime could explain the X-ray signal increase. Instead, using a laser beam with a realistic spot and phase profile shows that the focal plane position and the focusing of the laser also play an important role in the X-ray signal increase and allow to fully reproduce this trend. The reason explaining this trend is the improvement of the laser spot homogeneity when the focal plane is moved further inside the plasma.

Including the experimental profile and phase of the laser leads to lower electron charges and energies involved during the acceleration and drastically decreases the number of emitted photons as well as the critical energy of the radiation spectrum. This yields better results: the number of photons is reduced by one order of magnitude and the critical energy of the X-ray and the electron charge for the shortest nozzle are now much closer to the experimental results. Besides phase effects could be an attempt to explain the fluctuation of the electrons emission direction in the experiments.

In addition, understanding the laser quality effects is of primary importance as the focusing process plays a major role in laser propagation, self-injection, but also phenomenon such as self-truncated ionization injection^33^. The importance of this process in LWFA leads to worst performances achieved with a degraded laser spot quality as was shown. Presently, the main solution to increase the characteristics of the output – electron charge or energy, number of emitted photons, critical energy of the X-ray – relies on the increase of the power of the laser. However this work suggests that improving the laser spot quality would also lead to an important benefit.

## Methods

### Electron beam detection and photon counting

The electron spectrometer used in the experiments consists of two consecutive permanent dipoles magnets that deflect electrons and a Lanex phosphor screen to convert the electron energy into light which is then imaged by a CCD camera^34^. To determine the number of emitted photons and the critical energy, X-ray spectra were measured by photon counting with a direct detection deep-depletion X-ray CCD. More details can be found in reference 31.

### Simulations

3D simulations are performed with the PIC code CALDER. The cell dimensions are 0.25 *c*/*ω*_0_ longitudinally and 4 *c*/*ω*_0_ in the transverse directions. To reduce the computational costs, a 1800 × 400 × 400 cells moving window was used and a pre-ionized plasma was used instead of neutral He atoms. X-ray emission is post-processed from the recorded trajectories of test electrons with the formula 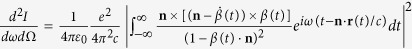, which gives the radiated energy 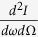 per frequency unit *dω* and per solid angle *d*Ω in the direction of observation **n**.

### Phase reconstruction

The two laser spots are measured in one single laser shot with the following technique. An imaging system allows to monitor the laser pulse spatial distribution at the laser nominal energy. It consists of a wedge that can be inserted in the laser beam optical path, followed by a zero degree high reflectivity mirror and a lens. The laser beam is then divided, using a beamsplitter mirror, toward two Charge-Coupled Device (CCD) camera. One CCD monitor the focal plane. The second one is imaging 5 mm after the focal plane. The imaging system magnification is ×6.4.

Gerchberg-Saxton algorithm is then run on these two spots. From an initial null wavefront and the experimental laser pulse spatial distribution in the focal plane, it performs a fast Fourier transform (FFT) to calculate the wavefront in the other plane. There it performs an FFT from the computed phase and the experimental laser spot in this plane to calculate a new wavefront in the initial plane. This cycle is iterated until GSA converges (≈20 iterations).

In the simulations with experimental spot and phase, the laser is initialized with both the transverse spot and wavefront calculated with the algorithm. The laser is launched from the left boundary of the simulation box. The amplitude of the field initialized at this boundary depends on the laser amplitude and phase computed by the GSA.

### Calculation of the number of photons

In the experiment, the photon distribution *d*^2^*N*/*dωd*Ω per frequency unit and per angle unit is calculated in the maximal emission direction. This distribution is then fitted with a synchrotron distribution 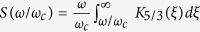 and finally integrated over the frequency and multiplied by an estimated angle 20 × 20 mrad^2^. The comparisons between experiment and simulation were done using the same method in the simulations. However a second and more accurate method was performed in the simulations to calculate *N*γ, which consists in the direct calculation of *d*^2^*N*/*dωd*Ω in each direction and its integration over the frequency and an angle 80 × 80 mrad^2^. The comparison of *N*_*γ*_ obtained by both methods suggests that the number of photons is systematically underestimated several times in the experiment (~3–4) due to the more simple calculation method, even if the trends remain the same.

## Additional Information

**How to cite this article**: Ferri, J. *et al*. Effect of experimental laser imperfections on laser wakefield acceleration and betatron source. *Sci. Rep.* **6**, 27846; doi: 10.1038/srep27846 (2016).

## Figures and Tables

**Table 1:** Experimental data.

**nozzle**	**density**	**plateau**	**ramp**	**focal plane**	***Q*** **[pC]**	***E***_***max***_ **[MeV]**	***N***_***γ***_	***E***_***c***_ **[keV]**
3 mm	6.2 × 10^18^ cm^−3^	3.1 mm	0.5 mm	−0.5 mm	202 ± 60	224 ± 19	1.8 ± 0.3 × 10^8^	12.3 ± 0.7
5 mm	6.4 × 10^18^ cm^−3^	5.6 mm	0.5 mm	0.3 mm	230 ± 57	250 ± 18	2.5 ± 0.9 × 10^8^	12.3 ± 0.9
7 mm	5.8 × 10^18^ cm^−3^	7.5 mm	0.65 mm	2 mm	303 ± 93	348 ± 31	4.9 ± 1.8 × 10^8^	15 ± 0.9

The length of the ramp is the same for both the entrance and exit ramp. The position of the focal plane is given relatively to the beginning of the plateau – minus means that the focal plane is before the beginning of the plateau. The electron charge *Q* > 140 MeV, the maximal energy of the electrons *E*_*max*_, the number of photons *N*_*γ*_ and the X_*-*_ray critical energy *E*_*c*_ are averaged over ten shots. The value indicated with ± denotes the standard deviation.

**Table 2:** Number of photons *N*_*γ*_ emitted in the interval 0–30 keV in the CALDER (3D) simulations.

**nozzle**	**3 mm**	**5 mm**	**7 mm**
gaussian	5.1 × 10^9^	7.2 × 10^9^	4.5 × 10^9^
experimental spot	1.8 × 10^9^	1.6 × 10^9^	2.4 × 10^9^
experimental spot and phase	8.5 × 10^8^	1.3 × 10^9^	2.0 × 10^9^
